# From the Carp Gut to Plastic Solutions: *Hafnia* Strain from *Cyprinus carpio* Demonstrates Robust Degradation of Synthetic Polymers

**DOI:** 10.3390/microorganisms14051101

**Published:** 2026-05-13

**Authors:** Mina Popovic, Boris Rajcic, Neveka Rajic

**Affiliations:** 1 Faculty of Ecology and Environmental Protection, University Union-Nikola Tesla, Cara Dusana 62-64, 11158 Belgrade, Serbia; nrajic@unionnikolatesla.edu.rs; 2 Institute of General and Physical Chemistry, University of Belgrade, Studentski trg 12/V, 11158 Belgrade, Serbia; brajcic@iofh.bg.ac.rs

**Keywords:** biodegradation, *Hafnia paralvei*, polyethylene, first-order kinetics, thermal analysis, Fourier-Transform Infrared (FTIR) spectroscopy, plastic pollution

## Abstract

The accumulation of polyethylene (PE) in aquatic ecosystems represents a significant environmental challenge due to the polymer’s high molecular weight and chemical stability. This study investigates the biodegradation potential of *Hafnia paralvei* UUNT_MP29, a bacterial strain isolated from the gut of common carp (*Cyprinus carpio*), for low-density polyethylene (LDPE). Initial screening on LDPE-emulsified agar confirmed extracellular enzymatic activity through the formation of distinct clear zones. Quantitative analysis showed a cumulative mass loss of 24.10% by Day 16, with the most intensive degradation occurring between Days 4 and 8, which closely correlated with maximum bacterial count (CFU/mL). Kinetic modeling indicated that the degradation followed a first-order rate law (R^2^ = 0.9269), with a rate constant (k) of 0.2991 days^−1^ and a remarkably short half-life (t_1/2_) of 2.32 days. Structural characterization via FTIR spectroscopy demonstrated oxidative transformation, evidenced by a reduction in sp^3^ C-H stretching and the emergence of C-O/C-O-C functional groups. SEM micrographs further confirmed extensive bio-deterioration, including surface pitting and macroscale erosion. Thermal analysis (TGA/DTG) supported these findings, showing a significant 10.95 °C decrease in the maximum degradation temperature (T_max_), indicating a reduction in polymer chain length. These results suggest that *H. paralvei* UUNT_MP29 is a highly efficient agent for the rapid breakdown of polyethylene and highlight the potential of aquatic gut microbiota as reservoirs for plastic-degrading biotechnologies.

## 1. Introduction

Polyethylene (PE) is the most widely utilized synthetic plastic globally, yet its profound environmental dilemma lies in its chemical stability. The same durability that makes PE indispensable to modern industry renders it exceptionally resistant to natural degradation [1,2,3]. Consequently, PE has become a ubiquitous and persistent pollutant, necessitating the development of sustainable remediation strategies. Among these, microbial degradation has emerged as a promising, eco-friendly, and cost-effective approach for managing plastic waste at scale [4].

The inherent resistance of PE to biodegradation stems from its molecular architecture. The polymer consists of high-molecular-weight, hydrophobic carbon chains linked by stable single bonds and lacks the hydrolyzable functional groups typically targeted by microbial enzymes. Furthermore, the semi-crystalline nature of PE—characterized by densely packed crystalline domains—limits the surface area available for enzymatic attack [5]. While abiotic pre-treatments such as photo-oxidation can introduce carbonyl groups that facilitate fragmentation, identifying microorganisms capable of directly attacking the polymer backbone remains a priority. Biological degradation typically proceeds through three stages: (1) biofilm formation on the hydrophobic surface; (2) biofragmentation via extracellular enzymes such as laccases and alkane hydroxylases; and (3) bioassimilation and mineralization of the resulting fragments [4].

Recent research has shifted focus toward specialized ecological niches, particularly the digestive tracts of various living organisms, as promising reservoirs for plastic-degrading microorganisms. The gut environment acts as a natural bioreactor where high microbial density, constant temperature, and the presence of endogenous surfactants or digestive enzymes may facilitate the breakdown of recalcitrant polymers. For instance, several studies have successfully isolated polyethylene-degrading bacteria from the larvae of *Tenebrio molitor* [6] and *Galleria mellonella* [6,7], identifying genera such as *Enterobacter* and *Bacillus* as key contributors to polymer fragmentation [8,9].

In aquatic environments, the intestinal tract of fish presents a particularly compelling yet under-explored frontier. Fish inhabiting plastic-polluted freshwater systems frequently ingest microplastics, exposing their gut microbiota to synthetic polymers as potential substrates. While the degradation of organic matter in the fish gut is well documented, the isolation of specific strains capable of actively degrading high-molecular-weight plastics such as LDPE remains limited. This represents a significant knowledge gap, as aquatic-derived strains may possess unique enzymatic adaptations optimized for submerged or anaerobic conditions.

Therefore, this study aims to isolate and characterize a robust plastic-degrading bacterial strain from the gut of common carp (*Cyprinus carpio*). We specifically investigate the ability of *Hafnia paralvei* UUNT_MP29 to utilize LDPE as a sole carbon source, providing a comprehensive analysis of the resulting chemical, physical, and thermal modifications to the polymer backbone to evaluate its potential for biotechnological environmental remediation.

Although taxa such as Rhodococcus ruber [10,11], *Bacillus* spp. [12,13], and *Pseudomonas* spp. [14,15] have demonstrated PE-degrading capabilities in laboratory settings, the search for novel, high-efficiency strains continues. Building on our recent findings that specific Hafnia strains effectively degrade polystyrene [16,17], this study explores the potential of a novel isolate, *Hafnia paralvei* UUNT_MP29, to degrade low-density polyethylene.

This strain was isolated from the gastrointestinal tract of the common carp (*Cyprinus carpio*), a unique biological niche that may function as a ‘natural bioreactor.’ As an omnivorous bottom-feeder, the carp constantly ingests various materials, including microplastics, alongside its natural diet. The distinct microbial consortium, neutral-to-alkaline pH gradient, and presence of diverse digestive co-substrates within the carp gut may exert selective pressure on resident microbiota, favoring the evolution of metabolic pathways capable of degrading chemically inert synthetic chains.

In this study, *Hafnia paralvei* UUNT_MP29 was evaluated for its capacity to utilize LDPE film as the sole carbon source over a 16-day incubation period. A multi-analytical approach was employed to characterize the degradation process, including viable cell counts (which was determined by the Colony-Forming Unit—CFU) to monitor biofilm development and surface colonization, and gravimetric analysis to quantify mass loss. Physical erosion and oxidative chemical transformations were corroborated using Scanning Electron Microscopy (SEM) and Fourier-Transform Infrared (FTIR) spectroscopy, respectively. Additionally, thermogravimetric analysis (TGA) was utilized to detect shifts in thermal stability, providing indirect evidence of polymer chain shortening. Based on the observed oxidative signatures and surface deterioration, the study discusses the possible involvement of extracellular enzyme systems. While the specific biochemical pathways remain to be definitively characterized, the results are consistent with the activity of oxygenase- or laccase-type enzymes, which are frequently associated with the biofragmentation of recalcitrant synthetic polymers in related microbial species.

## 2. Materials and Methods

### 2.1. Strain Characterization

The bacterial strain used in this study, *H. paralvei* UUNT_MP29, was isolated from the gastrointestinal tract of the common carp (*Cyprinus carpio*) and identified via 16S rRNA gene sequencing, as described by Dragačević et al. [16,17]. Species-level identification was confirmed by comparing the sequence with the NCBI GenBank database using the Basic Local Alignment Search Tool (BLAST+2.16.0; National Institutes of Health, Bethesda, MD, USA), which showed high similarity to known H. paralvei strains. This strain was selected for its previously reported potential for probiotic use and microplastic biodegradation. For the current study, the strain was maintained on nutrient agar at 30 °C.

#### 2.1.1. Enzymatic Screening

To further evaluate the isolate’s potential for specific polymer degradation, an initial screen was conducted using the “clear zone” method. Mineral salt medium (MSM) agar was supplemented with emulsified low-density polyethylene (LDPE), where the formation of distinct transparent halos around the colonies served as a functional indicator of extracellular enzymatic activity. These halos suggest the secretion of hydrolases or oxygenases capable of depolymerizing the LDPE substrate.

Although the genetic architecture of this strain was not mapped by Whole-Genome Sequencing (WGS) in this study, the observed degradation patterns and the well-documented metabolic versatility of the genus *Hafnia* suggest a robust enzymatic system. Based on chemical transformations identified via Fourier-Transform Infrared Spectroscopy (FTIR), the involvement of enzymes such as alkane hydroxylases, laccases, and lipases is hypothesized to be the primary driver of the observed biodegradation.

The degradation potential was monitored over a 16-day incubation period. In addition to inoculated samples, abiotic control samples consisting of sterile LDPE films in non-inoculated MSM were prepared to account for potential abiotic oxidation or hydrolysis. The MSM composition was (NH_4_)_2_SO_4_ (2.0 g/L), KH_2_PO_4_ (0.7 g/L), Na_2_HPO_4_·12H_2_O (1.25 g/L), MgSO_4_·7H_2_O (0.1 g/L), and trace elements. All chemicals were purchased from Sigma-Aldrich (St. Louis, MO, USA). Flasks containing 50 mg of LDPE film were maintained at 37 °C and 150 rpm.

To assess the effectiveness of the strains, the following analytical approach was employed:

- Viable Cell Counts: Biofilm formation was confirmed by monitoring colony-forming units (CFU/mL) to indicate the strain’s ability to utilize the polymer as a primary growth substrate.
- Gravimetric Analysis: Net weight loss was measured using an analytical balance (precision ±0.1 mg) to assess polymer mass removal.
- Surface and Structural Characterization: Scanning electron microscopy (SEM) visualized physical erosion, while FTIR detected the emergence of oxygen-containing functional groups.
- Thermal Stability (TGA): Thermogravimetric analysis tracked shifts in decomposition profiles; a decrease in the maximum degradation temperature (T_max_) served as a proxy for reduced molecular weight.

The 16-day period was chosen based on preliminary experiments showing that *H. paralvei* UUNT_MP29 reaches peak metabolic activity within the first week, aligning with established protocols [1,2].

Colonization density was monitored using the viable cell count method [18]. At intervals (Days 0, 4, 8, 12, and 16), LDPE films were rinsed with sterile phosphate-buffered saline (PBS, pH 7.4), placed in 10 mL of sterile saline (0.85% NaCl), and subjected to ultrasonic treatment (40 kHz for 5 min) followed by vortexing (2 min) to suspend the biofilm. Aliquots (100 µL) were spread onto MRS plates and incubated at 37 °C for 24 h. Results were converted from CFU/mL to CFU/cm^2^ by dividing the total number of recovered cells by the total surface area of the LDPE film (2 × length × width). All measurements were performed in triplicate and analyzed using Origin 2025b software (OriginLab, Northampton, MA, USA) to ensure statistical reliability.

#### 2.1.2. Screening for Polyethylene-Degrading Activity

The bacterial isolates were screened for low-density polyethylene (LDPE) degradation using the clear zone method on mineral salt medium (MSM) agar plates. Following a modified protocol based on Nademo et al. [19], the medium was prepared by incorporating 0.1% (*w*/*v*) emulsified LDPE into MSM supplemented with 1.5% (*w*/*v*) agar. Briefly, isolated colonies were initially cultured on nutrient agar, after which 100 μL of the standardized inoculum was applied to the LDPE-supplemented plates and incubated at 37 °C for up to 16 days. The plates were monitored regularly for the appearance of distinct transparent halos (clear zones) around the colonies. The formation of these zones confirmed the secretion of extracellular polymer-degrading enzymes and was used as the primary indicator of LDPE-degrading activity.

#### 2.1.3. Experimental Procedure and Weight Loss Calculation

LDPE film (60 µm thick), made of linear ethylene monomer chains (C_2_H_4_), was cut into 1 cm^2^ square strips. After a 16-day incubation, treated samples were immersed in distilled water for 4 h to remove the biofilm, then dried overnight at 60 °C. The samples were weighed using a four-digit analytical balance. To reduce experimental error, three control sets (parent LDPE and LDPE with biofilm) were processed alongside the treated samples. Percent weight loss was calculated according to Equation (1):

(1) Weight Loss (%) = (W_0_ − W_t_)/(W_0_) ∙ 100%

where W_0_ represents the initial weight and W_t_ represents the residual weight at time t.

##### Degradation Kinetics

To assess the degradation efficiency of polyethylene by *Hafnia* sp., the consumption rates were modeled using first-order kinetics. The natural logarithm of the residual polyethylene mass [ln(Mass)] was plotted against incubation time (days) to find the degradation rate constant (k). Linear regression was performed based on the integrated first-order rate equation:

(2) ln(M_t_) = ln(M_0_) − k∙t

where M_t_ is the mass of the treated sample at time t, and M_0_ is the initial mass. The rate constant (k) was obtained from the slope of the regression line. All statistical calculations and linear regressions were performed to assess the metabolic rate of the *Hafnia* strain in degrading the polymer substrate. The half-life (t_1/2_) of the polyethylene under these specific microbial conditions was calculated using the relationship t_1/2_ = ln(2)/k.

### 2.2. Instrumental Analysis

#### 2.2.1. Thermogravimetric Analysis (TGA)

To evaluate changes in thermal stability and decomposition kinetics, TGA was performed on an SDT-Q600 simultaneous TGA/DSC analyzer (TA Instruments, New Castle, DE, USA). Approximately 5–10 mg of residual LDPE film from both the control and Hafnia-treated samples was washed with distilled water and then dried to a constant weight. Samples were placed in an alumina crucible and heated from 25 °C to 600 °C at a steady rate of 10 °C/min. The analysis was conducted under a continuous nitrogen flow (50 mL/min) to monitor the thermal breakdown of the polymer backbone. The onset temperature (T_onset_) and the peak decomposition temperature (T_max_) were determined from the weight-loss curves and their corresponding derivative thermogravimetry (DTG) plots.

#### 2.2.2. FTIR Analysis

Analysis was performed using a Nicolet iS10 spectrometer (Thermo Scientific, Waltham, MA, USA) in attenuated total reflectance (ATR) mode. To evaluate the chemical modifications induced by the bacteria, pristine LDPE films incubated in sterile, non-inoculated MSM served as the negative control. These controls were used to account for any potential abiotic oxidation or environmental interference during the 16-day study. FTIR spectra were obtained at 4 cm^−1^ resolution covering a range of 4000–600 cm^−1^.

##### Quantitative Assessment via Carbonyl Index (CI)

To assess the extent of chemical oxidation caused by *Hafnia paralvei* UUNT_MP29, the Carbonyl Index (CI) was calculated as the ratio of the absorbance at the carbonyl band (A_1716_) to that at the methylene reference band (A_1452_). The conversion of Transmittance (%T) to Absorbance was performed in accordance with Equation (3)

(3) A = 2 − log_10_ (%T)

#### 2.2.3. SEM Analysis

The microstructure of the LDPE was examined using a JSM-6390 (JEOL, Tokyo, Japan) scanning electron microscope at 10 kV. Samples were evaluated at three points: initial (pristine LDPE) and after specific incubation periods. Before imaging, samples were fixed in 2.5% glutaraldehyde for 48 h and then dehydrated through a series of washes: 3 wt.% acetic acid; a 1:1 mixture of 3 wt.% acetic acid and 25 wt.% ethanol; a 1:1 mixture of 3 wt.% acetic acid and 50 wt.% ethanol; and finally, 70 wt.% ethanol. Samples were air-dried for at least 24 h, sputtered with a 10 nm layer of gold, and mounted on aluminum stubs for observation.

### 2.3. Statistical Analysis

All experiments were performed in triplicate (*n* = 3), and the results are expressed as the mean ± standard deviation. Statistical significance was evaluated using one-way ANOVA followed by Tukey’s post hoc test (*p* < 0.05) using Origin software (OriginLab, Northampton, MA, USA). Kinetic parameters and coefficients of determination (R^2^) were also calculated using Origin.

## 3. Results

### 3.1. Clear Zone Formation

**Figure 1:**
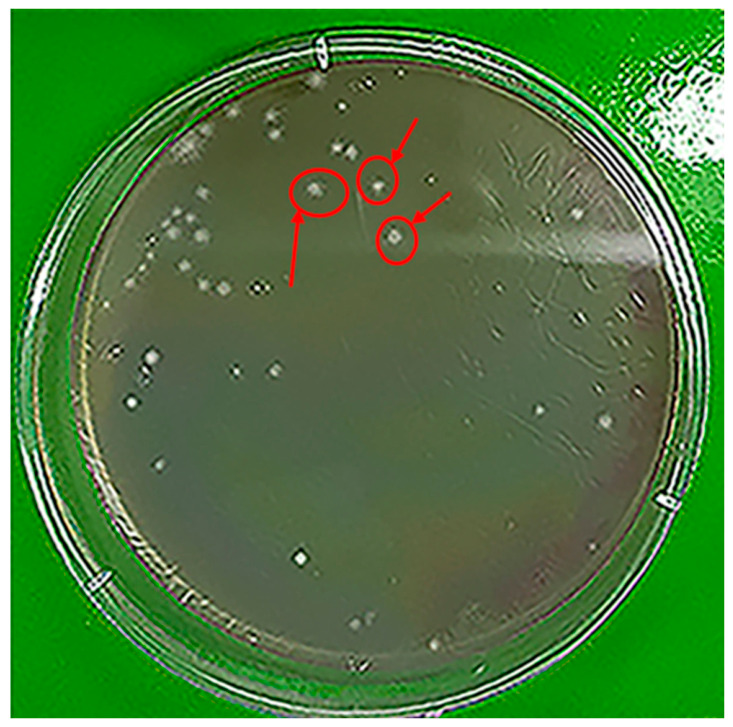
Clear zone formation and surface colonization of polyethylene by *Hafnia paralvei* UUNT_MP29. The red arrows show “clear zones” around the colonies. These zones indicate where the *Hafnia* strain has actively secreted enzymes to degrade the polyethylene substrate.

Initial screening on LDPE-emulsified agar plates revealed the formation of distinct transparent halos (clear zones) around *H. paralvei* UUNT_MP29 colonies after 16 days of incubation (Figure 1). The zones were characterized by a complete transition from the initial opaque white of the emulsified LDPE to transparency immediately surrounding the bacterial growth. No such zones were observed in the non-inoculated control plates.

### 3.2. Growth Dynamics and Gravimetric Analysis

**Figure 2:**
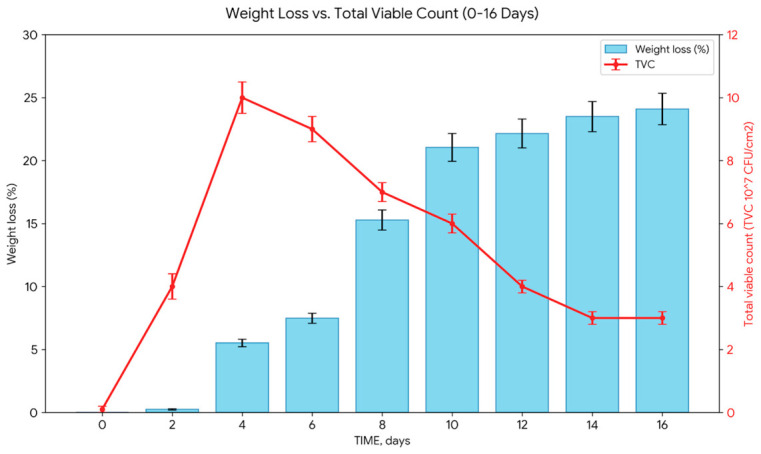
The data highlights a significant increase in cumulative biodegradation between Day 6 and Day 10, coinciding with the high-density phase of the bacterial population. The final cumulative weight loss reached 24.10% by Day 16.

**Figure 3:**
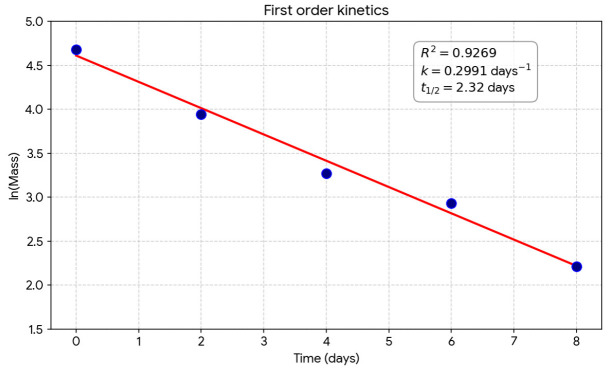
The degradation process was modeled using first-order kinetics (ln(M_t_/M_0_) = −k·t). The plot illustrates the linear relationship between the natural logarithm of residual mass and time (R^2^ = 0.9269), showing the rapid mass reduction observed during the first 8 days of treatment.

The biodegradation of LDPE by *H. paralvei* UUNT_MP29 followed a first-order rate law (R^2^ = 0.9269), with a calculated rate constant (k) of 0.2991 days^−1^ and a half-life (t_1/2_) of 2.32 days (Figure 2). Gravimetric analysis showed that the highest rate of mass loss occurred within the first 4 days. Concurrently, microbial populations reached a peak density of 10^8^–10^9^ CFU/mL by Day 4 and remained stable throughout the 16-day incubation period (Figure 3).

#### Degradation Kinetics

The gravimetric data for the 16-day incubation period of LDPE films with *H. paralvei* UUNT_MP29 are summarized in Table 1. The most significant mass loss occurred during the early stages of incubation, with the highest incremental biodegradation efficiency observed on Day 8. This rapid initial weight loss coincides with the exponential growth phase of the bacterial population, during which the accessibility of the amorphous regions of the LDPE is at its maximum. In contrast, abiotic controls showed no significant mass loss (<0.2%).

As incubation progressed, the rate of mass loss slowed, a “leveling off” attributed to the exhaustion of easily accessible amorphous segments and the potential accumulation of metabolic intermediates. Despite this transition, the cumulative mass loss was successfully fitted to a first-order kinetic model.

The logarithmic transformation of the gravimetric data produced a linear correlation (R^2^ = 0.9269), confirming that the degradation rate remains proportional to the remaining polymer mass. Although the calculated half-life (t_1/2_ = 2.32 days) primarily reflects the biofilm’s high-activity window, the consistency of the first-order fit suggests that the fundamental mechanism of chain scission remains active as the polymer’s crystalline fraction becomes the predominant substrate.

### 3.3. FTIR Analysis

**Figure 4:**
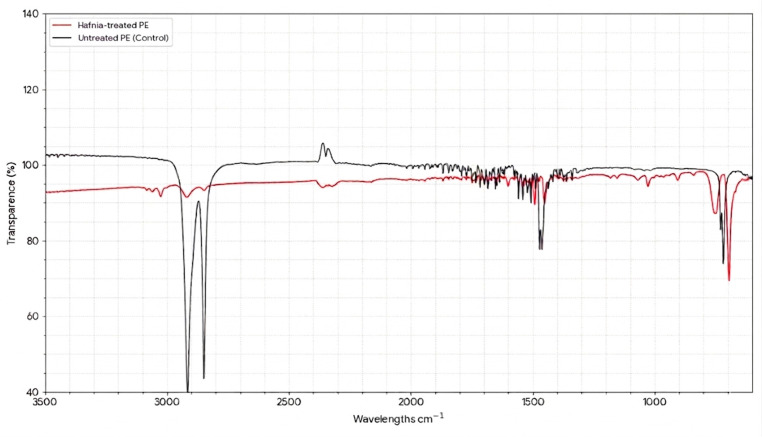
Comparative FTIR spectra of *Hafnia*-treated polyethylene (LDPE) and untreated LDPE (control). Control is in black, and the treated sample is in red.

The comparative FTIR spectra of treated LDPE and the sterile control are shown in Figure 4. The sterile control (negative control) displayed the characteristic spectrum of pristine polyethylene, with no detectable oxygen-containing functional groups, confirming that no abiotic oxidation occurred during the incubation period.

In contrast, the LDPE samples treated with H. paralvei UUNT_MP29 showed a significant decrease in the intensity of sp3 C-H stretching vibrations at 2914 and 2846 cm^−1^. Furthermore, new peaks emerged between 1200 and 1000 cm^−1^, corresponding to C-O and C-O-C ether/ester linkages, which were absent in the control sample. These chemical signatures provide direct evidence of microbially mediated oxidative cleavage of the polymer backbone. The Carbonyl Index (CI), calculated to quantify this oxidation, was 0.4594 for the treated samples, compared with a baseline of 0.0 for the controls.

### 3.4. SEM Analysis

**Figure 5:**
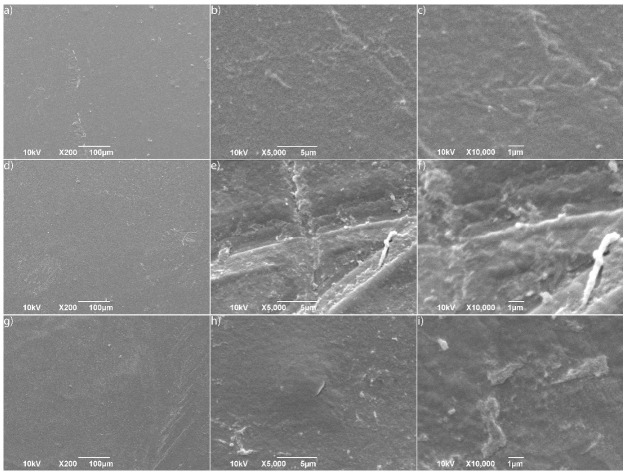
(**a**–**c**) pristine LDPE and (**d**–**f**) *Hafnia*-treated LDPE over 8 days, and (**g**–**i**) *Hafnia*-treated LDPE over 16 days under different magnifications (200–10,000×).

Scanning Electron Microscopy (SEM) was used to characterize the surface topography of the LDPE films. The pristine LDPE (control) displayed a smooth, continuous, and uniform surface even at 10,000× magnification, with no visible cracks or pits (Figure 5a–c).

Following 8 days of incubation with *H. paralvei* UUNT_MP29, the surface developed microcracks and distinct circular pits, with rod-shaped bacterial cells visible within an extracellular polymeric substance (EPS) matrix (Figure 5d–f). By Day 16, the deterioration advanced to deep, irregular fractures and noticeable thinning of the film. The micro-cracks merged into larger, interconnected fissures, and under 10,000× magnification, the polyethylene matrix exhibited a “spongy” or fibrous appearance (Figure 5g–i).

### 3.5. Thermal Analysis

**Figure 6:**
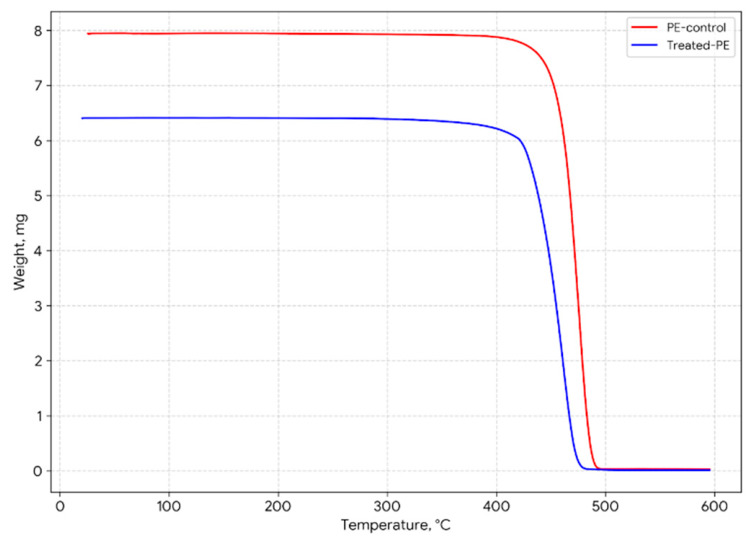
TGA curves of pristine polyethylene (control) and *Hafnia*-treated polyethylene.

The thermal stability of the LDPE films before and after treatment was evaluated using TGA and DTG analysis. The TGA curves (Figure 6) show a leftward shift for the treated samples compared to the control. The onset temperature T_onset_) decreased from 459.17 °C in the control to 436.23 °C in the samples treated with *H. paralvei* UUNT_MP29. Similarly, the maximum degradation temperature (T_max_) decreased from 474.18 °C to 463.23 °C.

**Figure 7:**
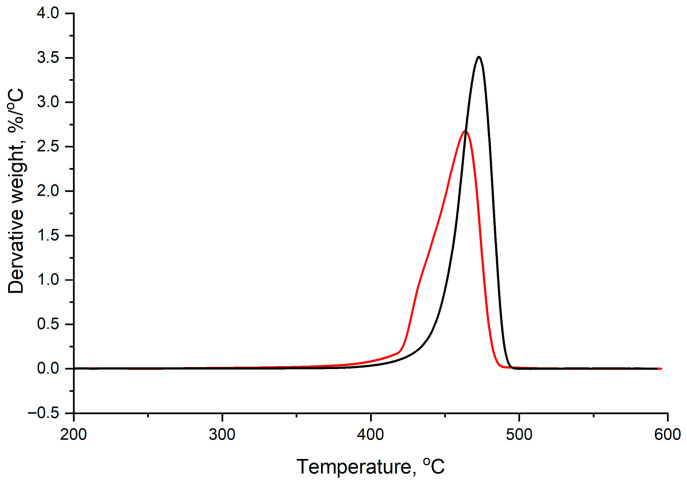
DTG curves of pristine polyethylene (control, black) and *Hafnia*-treated polyethylene (red).

The derivative thermogravimetric (DTG) curves (Figure 7) showed a corresponding shift in the peak decomposition rate toward lower temperatures.

Comparative results are provided in Table 2.

## 4. Discussion

### 4.1. The Carp Gut as a Specialized Bioreactor for Plastic Degradation

The degradation efficiency of *H. paralvei* UUNT_MP29 observed in this study (21.0% mass loss by Day 4) ranks among the highest reported for gut-derived bacterial isolates. Compared with the work of Perez Carpio et al. [20], which used *Galleria mellonella* microbiota, our strain exhibited a significantly faster onset of oxidative transformation. While many studies report incubation periods of 30 to 60 days to achieve similar mass loss, the rapid kinetics observed here (t_1/2_ = 2.32 days) suggest that the fish gut environment may harbor highly specialized strains optimized for the rapid processing of ingested polymers.

The 10.95 °C decrease in T_max_ identified via TGA is a critical indicator of depolymerization. This shift is more pronounced than that reported by Skariyachan et al. [21], where *Bacillus* and *Pseudomonas* consortia achieved shifts of only 5–7 °C over 120 days. The substantial reduction in thermal stability in our study confirms that *H. paralvei* UUNT_MP29 does not merely colonize the surface but actively cleaves the high-molecular-weight polyethylene backbone into shorter hydrocarbon fragments.

Furthermore, the FTIR signatures—specifically the emergence of C-O and C-O-C groups—align with the metabolic pathways proposed for other proteobacteria. However, unlike the studies by Jeon and Kim [22], which focused on isolated alkane hydroxylases from *P. aeruginosa*, our results suggest a multi-enzymatic synergy. The “spongy” morphology seen in SEM micrographs indicates that the attack is concentrated on the amorphous regions, similar to the degradation patterns observed in mealworm-derived *Enterobacter* species.

The synergy between the host’s digestive surfactants and the microbial secretome likely explains this efficiency. The “priming” of the LDPE surface within the carp gut, followed by the robust biofilm formation and subsequent oxidative attack by *H. paralvei*, presents a potent biological mechanism for plastic breakdown that warrants further proteomic investigation.

### 4.2. Extracellular Enzymatic Activity and Surface Bio-Deterioration

The clear zones observed in Section 3 provide physical evidence of the degradative capacity of *H. paralvei* UUNT_MP29. This phenomenon suggests that the strain secretes extracellular enzymes that break down high-molecular-weight polyethylene chains into water-soluble intermediates, such as oligomers or monomers, which can then be bioassimilated. This extracellular process is likely mediated by specific enzymes, such as laccases or alkane hydroxylases, which initiate the oxidation of the carbon backbone. These enzymes introduce functional groups (e.g., carbonyl or hydroxyl) that reduce the polymer’s hydrophobicity, facilitating further attack.

Unlike soil-isolated strains such as *Bacillus* sp. PE4, which requires UV aging to show visible clearing [23], *H. paralvei* UUNT_MP29 demonstrated activity on pristine LDPE. This enzymatic capability is consistent with findings from other gut-derived isolates, such as *Enterobacter* and *Bacillus* species isolated from the larvae of *Tenebrio molitor* [6]. However, *H. paralvei* UUNT_MP29 exhibits a more rapid onset of activity, achieving visual depolymerization within 16 days without mechanical mastication or pre-treatment.

This activity drives significant structural changes. As the bacteria establish a stable biofilm—reflected in the rise in CFU/mL—they initiate a localized “etching” process. The resulting surface irregularities and deep pits observed via SEM (Figure 5) create a positive feedback loop by increasing the surface area available for further enzymatic attack. While many well-documented soil-derived strains like *Rhodococcus ruber* or *Pseudomonas* species primarily cause a “rugose” or roughened surface over 30 days [24,25], the “spongy” and highly porous morphology observed in this study within 16 days suggests a more aggressive penetration of the amorphous regions of the polymer matrix. This hollowing-out mechanism differentiates *H. paralvei* from slower-acting terrestrial isolates.

The verification of polyethylene biodegradation remains a significant challenge in environmental microbiology, as physical changes such as surface roughening do not always indicate true metabolic assimilation [26]. However, the combination of distinct clear zone formation and the deep “spongy” pits observed in this study suggests more than mere surface colonization. While recent studies have highlighted the slow nature of PE breakdown even with optimized consortia [27], *H. paralvei* UUNT_MP29 demonstrates a relatively rapid structural disintegration of pristine LDPE within 16 days.

#### Ecological Context: The Carp Gut as a Selective Micro-Environment

The observed efficiency of *H. paralvei* UUNT_MP29 can be attributed to evolutionary pressures within the carp intestinal tract. The dominance of Proteobacteria and Firmicutes in the gut of *Cyprinus carpio* reflects an environment optimized for rapid nutrient cycling [28]. As a filter-feeder, the carp naturally concentrates environmental microplastics, creating a “hotspot” for microbial colonization and interaction. While studies in other filter-feeders, such as the Silver Carp (*H. molitrix*), show gut dysbiosis upon exposure to plastic [29], *H. paralvei* UUNT_MP29 demonstrates a specialized adaptation. Its ability to aggressively hollow pristine LDPE suggests an enzymatic secretome uniquely adapted to the surfactant-rich, high-density microbial environment of the carp digestive tract, highlighting its potential as a high-efficiency bioremediation agent for aquatic environments.

### 4.3. Mechanistic Insights from FTIR and Thermal Analysis

The chemical transformations detected by FTIR spectroscopy provide critical clues about the underlying enzymatic mechanism. The observed decrease in the intensity of sp^3^ C-H stretching vibrations (2914 and 2846 cm^−1^), coupled with the emergence of oxygen-containing functional groups (C-O and C-O-C), indicates oxidative cleavage of the polymer backbone. Since the sterile control films remained chemically unchanged, these transformations are confirmed to be biologically mediated. Although genomic sequencing was not performed in this study, the basis for identifying the potential involvement of alkane hydroxylases, laccases, and lipases lies in the correlation between these chemical signatures and established microbial metabolic pathways. In related *Hafnia* and *Pseudomonas* species, the breakdown of long-chain hydrocarbons typically begins with alkane hydroxylases (such as the AlkB system), which hydroxylate the inert C-C backbone. This is followed by laccases, which generate reactive radicals that promote further chain scission and increase the polymer’s hydrophilicity. Additionally, the rapid “etching” and surface pitting observed via SEM suggest a synergistic role for lipases, which enhance the bioavailability of hydrophobic substrates at the polymer–biofilm interface. The shift toward oxygenated functional groups aligns with the pathways proposed by Jeon & Kim [21], where enzymes introduce oxygen into the hydrophobic polymer chain. However, while studies on *Pseudomonas aeruginosa* often report the formation of ketone carbonyl groups (approx. 1715 cm^−1^), our strain primarily showed increased C-O/C-O-C ether and ester linkages. This variation in the FTIR fingerprint suggests that *H. paralvei* may utilize a different enzymatic suite—potentially similar to the specialized peroxidases found in the salivary glands of the wax worm *Galleria mellonella* [6]—that targets different oxidative “attack points,” potentially prioritizing ether linkages over terminal ketones. The significant decrease in T_onset_ and T_max_ observed in the TGA results indicates a substantial reduction in the thermal stability of the LDPE films. This shift is a hallmark of polymer chain scission; as the high-molecular-weight chains are broken down into shorter fragments by the enzymatic activity of *H. paralvei* UUNT_MP29, less energy is required to volatilize the material. A 10.95 °C decrease in T_max_ in only 16 days is noteworthy compared with other bacterial systems; for instance, studies on *Bacillus* or *Staphylococcus* species often report marginal shifts (<5 °C) even after 60 days [26]. Our results are more comparable to the rapid thermal shifts seen in the gut microbiota of the mealworm (*Tenebrio molitor*), suggesting that *H. paralvei* UUNT_MP29 possesses a specialized capability to penetrate the crystalline–amorphous interface of the polymer. While direct molecular weight determination via GPC was not performed, this marked shift in the thermal decomposition profile (DTG), coupled with the emergence of oxygenated functional groups, serves as a reliable proxy for a reduction in polymer chain length. This internal evidence corroborates the “spongy” morphology observed via SEM, demonstrating that the strain induces deep structural disintegration rather than superficial erosion.

### 4.4. Growth Kinetics and Biodegradative Activity of H. paralvei UUNT_MP29

The kinetic parameters (k = 0.2991 days^−1^; t_1/2_ = 2.32 days) indicate that *H. paralvei* UUNT_MP29 is an exceptionally efficient degrader compared with terrestrial isolates. For instance, well-studied soil-derived strains like *Rhodococcus ruber* or *Bacillus* spp. typically exhibit much slower kinetics, often requiring months to achieve the mass loss observed here in just four days [28]. This rapid degradation during the initial “biofragmentation” stage suggests that this gut-derived strain is specialized for high-velocity polymer breakdown.

The ability of *H. paralvei* UUNT_MP29 to maintain high population densities (10^8^–10^9^ CFU/mL) while using LDPE as a primary carbon source indicates efficient metabolic conversion. The sustained CFU counts suggest the formation of a robust biofilm, which serves as a confined “reaction center”. In such a microenvironment, extracellular enzymes are concentrated at the polymer–microbe interface, minimizing enzyme diffusion into the bulk liquid and maximizing the uptake of released oligomers [29].

It should be noted that quantifying microbial populations within a biofilm presents inherent technical challenges. Determining CFU counts from a plastic surface requires the effective detachment of cells from the EPS matrix. In this study, we utilized a combination of mechanical agitation and surfactant treatment to maximize cell recovery. While CFU counts represent only viable, culturable cells and may theoretically underestimate the total population compared to molecular methods such as PCR, the high population densities recorded (10^8^–10^9^ CFU/mL) demonstrate robust, stable colonization of the LDPE. This stability is a key indicator that the biofilm has successfully established a confined microenvironment for enzymatic activity, thereby directly facilitating the observed gravimetric and structural changes.

The direct correlation between peak biomass and the maximum weight-loss rate supports the hypothesis that *H. paralvei* UUNT_MP29 does not merely tolerate polyethylene but actively uses it as an energy source. This metabolic efficiency has significant implications for freshwater ecosystems; specialized gut-derived bacteria could play a pivotal role in the natural attenuation of plastic pollution in environments where microplastic residence times are otherwise long. Future efforts to isolate specific laccases or peroxidases from this strain may provide a foundation for scalable, enzyme-based biotechnological solutions for waste.

Although the biodegradation kinetics observed here were modeled with a first-order rate law for comparative purposes, they likely reflect a complex, nonlinear biological induction phase. Because the bacteria were not pre-induced with polyethylene before the experiment, an initial lag phase is expected, during which the *H. paralvei* UUNT_MP29 cells sense the hydrophobic substrate and begin to transcribe specialized extracellular secretomes. The rapid increase in degradation rates observed after initial contact suggests that as CFU density increases and the biofilm matures, the local concentration of induced enzymes—such as alkane hydroxylases and laccases—reaches a critical threshold, thereby accelerating biofragmentation. This induction-driven acceleration is consistent with the high R^2^ value obtained for our kinetic model, which integrates these biological transitions into a robust overall rate constant.

### 4.5. Morphological Evidence of Biodegradation: SEM Analysis

The transition from a smooth surface to a highly compromised, porous structure provides direct physical evidence of *H. paralvei* UUNT_MP29’s degradative capacity [30]. The formation of circular pits at Day 8 suggests that extracellular enzymes are localized at specific attachment sites, creating “hot spots” of accelerated polymer chain scission. This localized etching creates a positive feedback loop: as the surface area increases through fracturing, more bacterial adhesion and enzymatic access are facilitated, leading to the substantial mass loss recorded in the gravimetric analysis.

The most striking observation is the “spongy” or fibrous morphology reached by Day 16. While many studies on *Pseudomonas* or *Bacillus* report a “rugose” or “roughened” surface—often requiring 30 to 60 days to manifest—the “spongy” architecture produced by *H. paralvei* within only 16 days is rarely documented in the literature [15]. This unique appearance suggests that this strain may preferentially penetrate and deplete the amorphous regions of the LDPE matrix, effectively hollowing out the polymer structure from within. This mechanism explains the significant decrease in thermal stability observed in the TGA results, as the loss of structural integrity in the amorphous regions facilitates earlier thermal decomposition. This aggressive internal penetration distinguishes gut-derived *H. paralvei* from typical soil-dwelling isolates, which primarily erode the polymer surface.

### 4.6. Ecological Significance of Gut-Derived Hafnia

The isolation of LDPE-degrading *H. paralvei* UUNT_MP29 from the gastrointestinal tract of *Cyprinus carpio* supports emerging theories regarding the “plastisphere” within aquatic vertebrates. As an omnivorous bottom-feeder, the common carp frequently encounters microplastics that sequester in benthic sediments. The ability of its gut-associated *Hafnia* strains to utilize LDPE as a primary carbon source suggests an adaptive metabolic response to the presence of synthetic polymers in the host’s diet.

The significant structural and thermal changes observed via FTIR and TGA indicate a multi-stage degradation process, likely driven by a synergy between the host’s internal environment and microbial enzymatic activity. It is probable that an initial “priming” effect—potentially involving the fish’s own digestive enzymes or bile surfactants—increases the polymer’s surface hydrophilicity. This modification facilitates microbial colonization and the formation of a stable biofilm. Once established, *Hafnia*-secreted oxidases and hydrolases target the more accessible amorphous regions of the LDPE matrix, as evidenced by the “spongy” morphology seen in SEM. This targeted attack weakens the polymer backbone and reduces overall crystallinity, a shift reflected in the marked decrease in thermal stability.

### 4.7. Comparison with Other Gut-Derived Plastic Degraders

The discovery of LDPE-degrading *Hafnia* in *C. carpio* mirrors recent breakthroughs in insect-based bioremediation. To date, the most prominent models of gut-mediated plastic degradation involve the larvae of the wax moth (*Galleria mellonella*) and mealworms (*Tenebrio molitor*) [6,31]. In these systems, specialized microbiota—including *Enterobacter* and *Bacillus*—metabolize polyethylene through a documented synergy of mechanical mastication and enzymatic processes.

As summarized in Table 3, *H. paralvei* UUNT_MP29 demonstrates significantly greater efficiency than these established systems. While the *G. mellonella* larval system remains exceptionally fast due to unique salivary enzymatic priming) [31], and *T. molitor* can achieve a high cumulative mass reduction (up to 69.7%) over 56 days [32], our purely bacterial isolate achieved a 21.0% weight loss in just 16 days (Table 2).

When normalized to time, *H. paralvei* UUNT_MP29 exhibits a degradation rate of approximately 1.31% per day. This rate surpasses the 0.87–1.24% daily rates observed in complex *Tenebrio larval* systems and significantly exceeds established soil-derived strains, such as *Rhodococcus* ruber (0.26% per day) and *Bacillus* species (0.17% per day). The ability to achieve rapid biofragmentation without physical mastication suggests that the fish gut acts as a specialized “evolutionary incubator,” selecting for bacterial strains with robust, independent secretomes. This is further corroborated by the high Carbonyl Index (CI) of 0.4594, a chemical marker confirming successful oxidative degradation driven by the strain’s specialized enzymatic repertoire.

Unlike the rapid transit times typical of insect larvae, the digestive system of *Cyprinus carpio* creates a specialized, stable environment for cultivating plastic-degrading microbiota. This efficiency is likely driven by two main mechanisms: (1) Enzymatic Priming: the carp gut naturally maintains high levels of endogenous proteases, amylases, and lipases [33]. *Hafnia* strains isolated from this environment thrive in a “pre-digestive” setting where host enzymes may enhance polymer bioavailability by softening surfaces or making chemical changes. (2) Synergistic Degradation: the notable shift in T_max_ observed through TGA indicates that carp-derived *Hafnia* strains are highly effective at promoting chain scission. While this is similar to the degradation efficiency of *Enterobacter asburiae* in mealworm guts, these strains are specially adapted to the high-moisture, aquatic conditions typical of freshwater hosts.

#### Implications for Aquatic Bio-Cycling

While insect-based studies focus on localized terrestrial degradation, this research indicates that a similar “bio-cycling” of microplastics occurs within freshwater ecosystems. The detection of *Hafnia* in the carp gut demonstrates that these fish are not merely passive victims of plastic pollution; they harbor a microbiome that actively adapts to utilize synthetic polymers as a carbon source. This prompts important questions about the metabolic costs to the host and the potential to harness these “environmentally trained” bacteria for industrial-scale bioreactors.

### 4.8. Comparative Analysis of Gut-Derived Plastic Degraders

To evaluate the effectiveness of *H. paralvei* UUNT_MP29, a comparative analysis was conducted against established gut-derived microbial models (Table 4). While most current studies focus on terrestrial insect larvae—such as the yellow mealworm (*Tenebrio molitor*) and the greater wax moth (*Galleria mellonella*)—our findings expand the scope of gut-mediated bioremediation to aquatic vertebrates.

### 4.9. Substrate Specificity: LDPE vs. Polystyrene

Finally, previous observations from our research group indicate that *H. paralvei* UUNT_MP29 also exhibits degradative activity toward polystyrene (PS). The ability of this strain to process both polymers is significant; however, the “rapid breakdown” observed in this study is particularly pronounced for LDPE.

Typically, strains isolated from the gut of *C. carpio* are well-suited for breaking down long-chain hydrocarbons. The linear structure of polyethylene enables extracellular enzymes from *H. paralvei* to align more effectively with the carbon backbone once a stable biofilm forms. In contrast, the bulky phenyl rings in the polystyrene molecular structure create a steric barrier that hinders enzyme access to the main chain. This structural difference explains the slower mass loss seen in polystyrene compared to the high efficiency observed here for LDPE.

### 4.10. Limitations and Future Perspectives

We acknowledge that the absence of Whole-Genome Sequencing (WGS) and direct molecular weight analysis limits the ability to fully resolve the metabolic pathways of *H. paralvei* UUNT_MP29. Future investigations will prioritize WGS to identify the specific genetic clusters responsible for producing alkane hydroxylases and laccases. This genomic mapping, combined with chromatographic molecular weight analysis, will improve the evaluation of the organism’s efficacy and provide a definitive blueprint for its application in scalable biotechnological remediation.

## 5. Conclusions

This study establishes that *Hafnia paralvei* UUNT_MP29, a gut-associated isolate from *Cyprinus carpio*, is a highly efficient biological agent for degrading low-density polyethylene (LDPE). The findings confirm that the specialized environment of the aquatic vertebrate gut serves as a reservoir for microbiota with unique metabolic adaptations that can overcome the chemical stability of synthetic polymers.

The novelty of this work lies in the identification of a non-canonical bacterial genus (*Hafnia*) that exhibits degradation kinetics significantly faster than those of traditional soil-dwelling isolates. While terrestrial strains often require months to initiate structural change, *H. paralvei* UUNT_MP29 demonstrates rapid biofragmentation and deep oxidative penetration, achieving a 24.10% mass loss within 16 days. The transition to a “spongy” polymer morphology—corroborated by a 10.95 °C decrease in thermal stability (T_max_) and significant oxidative functionalization—suggests a potent enzymatic suite that targets the amorphous regions of the polyethylene matrix more aggressively than previously documented bacterial models.

Practically, these results provide a blueprint for a “bioprospecting” strategy focused on aquatic gut microbiomes. The strain’s ability to maintain high metabolic activity while using LDPE as a primary carbon source makes it a prime candidate for the development of bio-augmented waste treatment systems. Theoretically, this research suggests that the carp gut’s digestive surfactants and neutral-to-alkaline conditions may “prime” these bacteria for high-efficiency polymer degradation. Future research incorporating Whole-Genome Sequencing (WGS) and the proteomic isolation of specific secretomes will be essential for elucidating the genetic basis of this efficiency and for creating scalable, enzyme-based solutions to mitigate the global microplastics crisis.

## Figures and Tables

**Table 1:** Gravimetric Analysis of LDPE Biodegradation by *H. paralvei* UUNT_MP29.

Time (Days)	* Cumulative Mass Loss (%)	Incremental Loss (%)	Residual Weight (mg) *
0	0.00	0.00	100.0 ± 0.00
2	0.24 ± 0.05	0.24	99.76 ± 0.05
4	5.52 ± 0.30	5.28	94.48 ± 0.30
6	7.48 ± 0.40	1.96	92.52 ± 0.40
8	15.29 ± 0.80	7.81	84.71 ± 0.80
10	21.05 ± 1.10	5.76	78.95 ± 1.10
12	22.15 ± 1.15	1.10	77.85 ± 1.15
14	23.50 ± 1.20	1.36	76.50 ± 1.20
16	24.10 ± 1.25	0.60	75.90 ± 1.25

* Values represent the mean of triplicate experiments ± standard deviation. Starting mass normalized to 100 mg.

**Table 2:** Comparative analysis of TGA curves.

Sample	T_5%_ (°C)	T_10%_ (°C)	T_onset_ (°C)	T_max_ (°C)
LDPE-control	439.32	450.23	459.17	474.18
LDPE-treated	415.94	427.98	436.23	463.23

**Table 3:** Comparative efficiency of various biological systems and bacterial strains in LDPE degradation.

System/Bacterial Strain	Incubation Time	Weight Loss (%)	Mechanism/Reference
*Tenebrio molitor*	56–60 days	49–69.7%	A multi-step process that uses physical, chemical, and biological methods to break down the resistant polymer structure [32]
*Galleria mellonella* (Larvae)	36 h	55.6%	Salivary enzymes & gut microbiota synergy [8]
*Rhodococcus ruber*	30 days	8%	Biofilm formation & oxidative attack [10,11]
*Hafnia paralvei* UUNT_MP29	16 days	21%	Current Study: Rapid biofragmentation
*Bacillus* species	40–60 days	5.0–10.7%	Extracellular enzyme secretion [23]
*Pseudomonas* species	30 days	~5.0–7.0%	Hydrocarbon chain shortening [22]

**Table 4:** Comparative metabolic and environmental profiles of gut-derived polyethylene (LDPE) degrading systems.

Feature	Insect Gut Models (*Galleria mellonella*) *	Carp Gut *Hafnia* (This Study)
Primary Host Environment	Terrestrial/Chitinous	Aquatic/Mucosal
Typical Incubation	Short-term intestinal transit	16 days of controlled incubation
Primary Mechanism	Mechanical mastication + Microbial	Enzymatic biofragmentation
TGA Shift	Reported in polystyrene	Demonstrated for LDPE

* Adapted from [20].

## Data Availability

The original contributions presented in this study are included in the article. Further inquiries can be directed to the corresponding author.
